# Comparison between abdominal CT findings in intensive care unit (ICU) and non-ICU patients with Covid-19: experience from a tertiary care hospital

**DOI:** 10.4314/ahs.v23i4.10

**Published:** 2023-12

**Authors:** Himanshu Goyal, Binit Sureka, Nachiketa Mangaraj, Ashish Agarwal, Nikhil Kothari, MK Garg, Mithu Banerjee, Ashwini Agarwal, Pawan Garg, Taruna Yadav, Pushpinder Khera

**Affiliations:** Department of Diagnostic & Interventional Radiology, All India Institute of Medical Sciences - Jodhpur, India

**Keywords:** COVID-19, abdomen, imaging, CAM, CT

## Abstract

**Background:**

16-66% of COVID-19 positive patients may have abdominal symptoms and findings in abdominal CT. The yield of abdominal CT scan in patients having abdominal complaints is not known.

**Objectives:**

The aim of this study was to explore the various abdominal imaging manifestations of COVID-19 and COVID-19 associated Mucormycosis (CAM) and to identify the relevant clinical and laboratory features associated with severity of the symptoms.

**Methods:**

A retrospective single centre observational study was performed at a tertiary care hospital in Northwest India. All consecutive patients who had COVID positive RT-PCR report and had undergone abdominal Computed Tomography scan from March 2020 to November 2021 for various abdominal complaints were included. Demographic data, CT images and reports and all relevant lab parameters were collected.

**Results:**

Out of 75 patients, positive abdominal findings were seen in 65 patients. Hepatobiliary findings were seen in 41.3% (31 of 75; OR=1.9) and bowel abnormalities were found in 37.3% (28 of 75; OR=2.1) of COVID-19 patients. 7 patients who had renal infarcts or bowel ischemia were found to have COVID-19 associated Mucormycosis on histopathology.

**Conclusion:**

Chest CT severity score was positively correlated with most of the abdominal manifestations in patients requiring ICU admission. Elevated D-dimer levels were significantly associated with abdominal symptoms.

## Introduction

The coronavirus disease 2019 (COVID-19) pandemic that originated in 2019 still continues to present as an ongoing global threat in 2022. It is caused by an RNA virus belonging to the Corona viridae family^1^.

Common clinical features include fever, cough, and myalgia^1^,^2^. It can affect various other organs like lungs, kidney, heart, muscles and lead to serious complications like sepsis and disseminated intravascular coagulation (DIC)^2^. Although lung injury is most common, Gastrointestinal (GI) symptoms and complications are also been increasingly seen in these patients^3^-^7^. SARS-CoV-2 is transmitted via respiratory droplets^8^. COVID-19 associated Mucormycosis (CAM) has been unmasked in this ongoing pandemic and has significantly increased morbidity in patients. Endothelial injury, impaired blood glucose and increased free iron in COVID-19 patients favours growth of mucormycosis. Being an angio-invasive organism, it causes endothelium damage leading to vascular occlusion and end organ ischemic changes^9^,^10^. Abdominal organ involvement is rare, however, if it occurs, it has poor prognosis^10^,^11^.

COVID-19 patients with more severe gastrointestinal symptoms are prone to have worse outcome (more days of hospitalisation / death / need of mechanical ventilation)^12^,^13^. The aim of this study was to explore the various abdominal imaging manifestations of COVID-19 and CAM in an Indian population and to identify the relevant clinical and laboratory features associated with those findings as well as their association with severity of the symptoms.

## Materials and methods

### Patients

This was a retrospective single centre observational study performed at tertiary care hospital in Northwest India. Institutional review board approval was obtained for the study. Inclusion criteria were patients with positive RT-PCR report who had undergone abdominal Computed Tomography (CT) scan from (March 2020 to November 2021) for various abdominal complaints.

### Image acquisition

CT scans were performed on either Somatom Drive Dual Energy 256 Slice Multi Detector CT Scanner (Siemens Healthcare GmbH, Germany) or Somatom Definition Flash 128*2 Slice Multi Detector CT scanner (Siemens Healthcare GmbH, Germany) depending on the availability and policies of the department.

### Data collection and image analysis

Demographic data, CT images and reports and all relevant laboratory parameters were collected. All imaging studies were interpreted in a clinical setting by trained abdominal radiologist and two general radiologists.

### Statistical analysis

Demographic, clinical, and imaging data from patients admitted in the ICU were compared with those of non-ICU patients using univariable statistical tests, including independent t tests, Chi square tests, and Fisher exact tests. Odds Ratio was also calculated for all the abdominal manifestations of COVID-19 to suggest the likelihood in ICU and non-ICU patients.

## Results

### Patient characteristics and imaging

**Figure 1:**
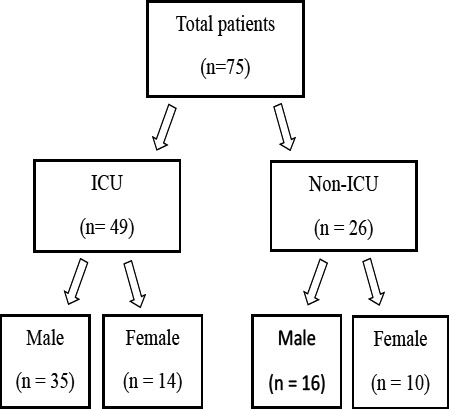
Flow chart demonstrating the patient distribution in our study

The flow of patients in our study is shown in Figure 1. A total of 75 patients underwent abdominal CT imaging in our institution during the study period, and of these, 49 patients (65%) were admitted to the ICU. Abdominal imaging findings were more common in patients admitted in ICU than non-ICU patients. Table 1 enumerates the indications for doing CT scan of abdomen for the patients with various complaints. The most common indications for CT were abdominal pain followed by abdominal distension.

**Table 1:** Summary of indications for CT Abdomen in COVID-19

Indications	Number of patients (n= 75)
Gastrointestinal system	53 (70.6)
Abdominal pain	52
Abdominal distension	10
Melena	4
Vomiting	1
Excretory system	5 (6.7)
Decrease urine output	5
Pus cell in urine	1
Respiratory system	3 (4)
Poor saturation	2
Hemoptysis	1
Others	14 (18.7)
Metastatic workup	5
Sepsis	3
Deranged LFT	2
Fever	2
Post abdominal surgery	2

### CT findings

**Figure 2:**
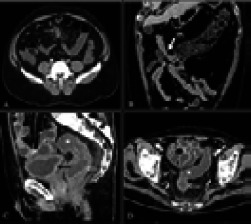
(A) Axial CECT abdomen image showing ileal thickening (thin white arrow) with surrounding mesenteric fat stranding in case of 52-year male with diffuse abdominal pain. (B) Coronal CECT image showing long segment thickening of proximal transverse colon (thick white arrow) in a 65-year-old male with melena. (C and D) Sagittal and axial sections of CECT abdomen in a 55-year-old male shows diffuse rectal wall thickening and fluid filled colon (white asterisks). Diffusely thickened urinary bladder wall is also seen (black arrow)

Table 2 illustrates the list of various abdominal imaging findings. Out of 75 patients, positive abdominal findings were seen in 65 patients. Hepatobiliary manifestations were the most common abdominal findings, seen in the form of hepatomegaly (25 out of 75) and hepatic steatosis (19 out of 75). One patient showed features suggesting ascending cholangitis. Bowel abnormalities were the second most significant abdominal findings, found in 37.3% (28 of 75) of abdominal CT images and were associated with ICU admission (OR= 2.1; p = .21). Bowel wall thickening (bowel wall thickening on CT was considered if single wall thickness is >3 mm in distended loops and >5mm in collapsed loops)^7^ was identified on 24% (18 of 75) of CT images (Figure 2) and included small-bowel thickening (n = 13) and colorectal thickening (n = 11). Among these 18 patients, 6 patients had both small and large bowel thickening. Bowel wall ischemia was seen in 12 of 75 patients (16%) and showed a significant preponderance to patients admitted in ICU than non-ICU patients (p=0.04; OR=7.2). Vascular thrombosis (SMA, SMV and their branches) was seen in 8 of the 12 cases.

**Table 2:** Summary of CT abdominal findings in ICU & Non-ICU patients with COVID-19

Parameter	ICU (49)	Non-ICU (26)	p value	Odds ratio
Liver findings	23	8	0.22	1.9
• Hepatomegaly	19	6	0.21	2.1
• Hepatic steatosis	13	6	0.78	1.2
• Periportal edema	1	2	0.27	0.25
Gastrointestinal tract findings	21	7	0.21	2.1
• Mural thickening	14	4	0.26	2.2
➢ Small bowel thickening	10	3	0.52	1.9
➢ Large bowel thickening	9	2	0.31	2.7
• Bowel wall ischemic changes	11	1	0.048	7.2
➢ Reduced enhancement	11	1	0.048	7.2
➢ Imperceptible thin wall	6	1	0.41	3.48
➢ Perforation	5	0	0.16	
➢ Pneumatosis intestinalis	3	0	0.54	
• Small and large bowel Obstruction	9	3	0.52	1.75
➢ Ileus	3	1	1	1.63
• Fluid filled colon	7	1	0.24	4.17
• Retained colonic stool	3	2	1	0.84
• Acute appendicitis	1	0	1	
Biliary tree finding	7	3	1	1.3
• GB distension	3	2	1	0.78
• Mural edema/wall thickening	2	1	1	1.06
• Biliary dilation	2	0	0.54	
• Cholangitis	1	0	1	
Pancreatic findings	4	1	0.65	2.22
• Acute interstitial pancreatitis	2	1	1	1.06
• Acute necrotizing pancreatitis	2	0	0.54	
Splenic findings	9	4	1	1.23
• Splenomegaly	6	3	1	1.07
• Splenic infarcts	3	1	1	1.63
Excretory system findings	17	6	0.43	1.77
• Peri nephric fat stranding	17	6	0.43	1.77
• Renal infarcts	8	1	0.15	4.8
• Urinary bladder findings	5	1	0.65	2.84
➢ Peri-vesical fat stranding	3	1	1	1.63
➢ Mural thickening	2	1	1	1.06
Vascular findings	14	2	0.04	4.8
• Arterial occlusion	13	2	0.07	4.3
• Venous thrombosis	4	0	0.29	
Peritoneal findings:	18	8	0.8	1.3
• Mesenteric fat stranding	18	8	0.8	1.3
• Ascites	17	6	0.8	1.3
• Peritonitis	5	1	0.65	2.84
• Free air	5	0	0.16	
COVID-19 associated Mucormycosis	6	1	0.43	3.48
No significant abdominal findings	5	5	0.3	0.48

**Figure 3:**
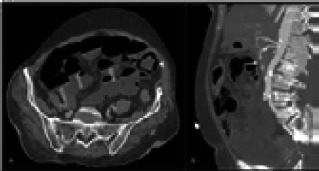
70-year-female COVID positive with abdominal pain and distension. Lab parameters showed raised TLC, CRP & D-dimer. (A) Axial post-contrast CT shows dilated bowel loops with multiple air-fluid levels (white arrows) and presence of intramural air (arrow head) suggestive of pneumatosis intestinalis. (B) Sagittal image in arterial phase shows non opacification of SMA (white arrow) suggestive of thrombosis

**Figure 4:**
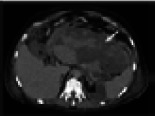
40-year-male COVID positive with severe abdominal pain: CT scan shows bulky pancreas which is replaced by necrotic material suggestive of necrotizing pancreatitis (arrows)

**Figure 5:**
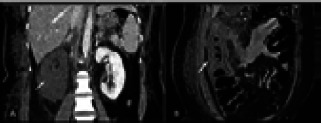
36 years non diabetic female presented with severe abdominal pain with raised TLC, serum creatinine and D-dimer. (A and B) Coronal CECT sections show complete non- enhancement of right kidney (solid white arrow) with perinephric fat stranding and long-segment thickening and hypo-enhancement of ascending colon (solid white arrow) with peri-colonic fat stranding suggesting ischemic changes. Histopathology done on operative specimen showed angio-invasive fungal profiles suggesting mucormycosis

The most common finding associated with ischemia was reduced wall enhancement in post-contrast study. Pneumatosis intestinalis was identified in 3 cases (Figure 3). Five patients in ICU (10%) had perforated small bowel (confirmed on CT scan), as evidenced by intraperitoneal free air. Patients admitted in the ICU were more likely to have this finding as compared to non-ICU patients (65% vs 23%, p = .04). Dilated bowel loops were noted in 16% (12 of 75) of cases. Dilated large bowel loops with faecal loading/air-fluid level was noted in 5 patients due to paralytic ileus. Acute pancreatitis was seen in 5 cases (Figure 4). Twelve patients (16% of CT findings) had evidence of acute infarction in at least one solid organ (renal, splenic) with majority in ICU (OR=3p= 0.1). Vascular thrombotic occlusion (SMA, SMV and their branches) was seen in 21% of patients (16 out of 75) with a significantly increased likelihood in ICU patients compared to non-ICU patients (OR=4.3, p=0.04). 7 patients who had renal infarcts or bowel ischemia were found to have COVID-19 associated mucormycosis (Figure 5) on histopathology which is enumerated in Table 3. Other abdominal CT findings included perinephric & perivesical fat stranding with thickened urinary bladder wall suggesting inflammation. Mesenteric findings were seen in 26 patients in the form of mesenteric fat stranding, ascites and peritoneal air.

**Table 3:** Summary of findings of COVID-19 associated Mucormycosis

COVID-19 associated Mucormycosis	Number of patients (n=7)
Renal infarct	6
Bowel ischemic changes	5
Arterial occlusion	5
Venous thrombosis	2

## Discussion

COVID-19 is a multisystem disease, most commonly affecting the respiratory system. Abdominal manifestations involving the bowel and liver have been reported frequently in patients with COVID-19 3-7.

In the last two years, since the beginning of pandemic, a number of studies on abdominal imaging in COVID-19 have been performed in various institutions across the world with varying sample sizes. A list of the various original research works with at least 10 or more sample size with their study characteristics and conclusions is enumerated in Table 4.

**Table 4:** Summary of research studies on abdominal CT findings in COVID-19

Author	Year	Sample size	Conclusion
Goldberg-Stein S et al 6	2020	141	57% cases had abdominal findings; bowel wall mural thickening was the most common finding.
Bhayana R et al 7	2020	42	43% cases had abdominal findings; bowel abnormalities and gallbladder bile stasis were common findings in ICU patients.
Barkmeier DT et al 14	2020	43	16% cases had abdominal findings; bowel inflammatory change was the most common findings.
Abdelmohsen MA et al^18^	2020	30	30% cases had abdominal findings; Ischemic and inflammatory bowel changes were more common.
Shiralkar K et al^43^	2020	10	42% cases had abdominal findings; Gastric and bowel wall abnormalities were more common.
Lei P et al^31^	2020	115	26% cases had liver findings; Liver hypodensity and pericholecystic fat stranding were commonly seen.
Horvat N et al^12^	2021	81	55% cases had abdominal findings; Intestinal distension was the most common finding.
Funt S. A. et al^19^	2021	597	45% cases had abdominal findings; Inflammatory findings were more common.
Patel RK et al^17^	2021	15	66.7% cases had abdominal findings; Biliary stasis and bowel involvement were the common findings in COVID-19.
Taya M et al^24^	2021	63	22.2% cases had abdominal findings; Thickening of the bowel or fluid-filled colon was commonly seen.
Tirumani SH et al^20^	2021	72	18.1% cases had abdominal findings; Fluid-filled colon was most common finding.
Vadvala HV et al^25^	2021	45	46.7% cases had abdominal findings; Cholestasis, hematoma and vascular occlusion were seen.

Bowel abnormalities are quite common in COVID-19 patients and mainly seen as mural thickening and bowel ischemic changes^6^,^7^,^12^-^24^. Possible explanations for the bowel findings in patients with COVID-19 is high concentration of ACE-2 surface receptors in enterocytes of small intestine, and vascular endothelium, making them susceptible for direct viral injury^7^. In our study, bowel wall thickening was the most common bowel abnormality. This was concordant with many past studies^6^,^14^-^17^,^19^,^20^,^22^,^24^. Small bowel thickening (n=13) was seen in more patients than colorectal thickening (n=11). A systematic review of 47 studies done by Ojha V et al. concluded showed similar results^23^. Bowel wall ischemic changes were more common in ICU patients and patients with higher CT severity index (p=0.01)^7^,^12^,^25^. Bowel perforation leading to pneumoperitoneum was seen in 5 patients, further increasing morbidity and mortality. Vascular occlusion was seen in 62% patients with bowel ischemia, in contrast to study done by Ojha et al that described non occlusive mesenteric ischemia as the most common pattern of bowel involvement^23^. In our study, raised D-dimer was found to be associated with ischemic bowel findings. Hypercoagulable state can significantly increase morbidity in COVID-19 by causing vascular occlusion leading to end organ ischemia and can be evaluated by serial monitoring of D-dimer^26^,^27^. Small and large bowel obstruction was seen in 12 patients (16%) secondary to mural thickening, bowel ischemia or functional ileus. 4 out of 12 patients did not have any transition point suggesting ileus that can be due to dysregulation of autonomic control of colonic motility^28^,^29^. In our cohort, most of the patients who had bowel involvement presented with abdominal pain. 2 out of 4 patients who had melena were found to have bowel wall thickening while 1 patient had bowel ischemia. Fluid filled colon was seen in only 8 patients that signifies diarrhoea in COVID-19 patients. This is in concordance with study done by Goldstein et al but in contrast with that of Bhayana R et al^6^,^7^. Since bowel abnormalities have significant association with severe pulmonary disease (p=0.01), if critically ill patients develop abdominal symptoms, then they should be promptly evaluated and an abdominal CT scan is invaluable in identifying the possible cause.

Hepatobiliary system involvement was the most common abdominal finding (after bowel abnormalities) in our cohort in contrast to some past studies. Hepatomegaly was seen in 33% of patients which could be due to direct viral mediated hepatitis^30^. Similarly hepatic steatosis was also seen in significant number of patients. Both these findings are in concordance with study done by Lei et al^31^. Hepatic involvement showed no significant difference between ICU and non-ICU patients (p=022). Gall bladder distension suggesting cholestasis and gall bladder wall thickening was also seen in our cohort similar to past studies^6^,^7^. One case of cholangitis was seen in our study in a patient with severe abdominal pain, high grade fever and raised ALP. The proposed mechanism for biliary tract inflammation includes direct viral cytopathic injury to cholangiocytes, microthrombi formation in the biliary tree vasculature, decreased splanchnic circulation due to high positive pressure ventilation or autoimmune injury. Pre-existing hepatobiliary disease further increases the risk^32^.

Incidence of pancreatic involvement in our cohort was similar to systemic review done by Singh P et al^16^. 5 patients showed features consistent with pancreatitis. Pancreatic involvement can be due to direct viral injury as ACE 2 is expressed on both exocrine and endocrine cells, host immune mediated cytokine response, dehydration or drug induced^30^,^33^-^35^. In our cohort, splenic abnormality was seen in 13 patients in the form of splenomegaly (10 cases) and splenic infarcts (4 cases). Renal injury in COVID-19 is well documented - possible mechanism for renal involvement in COVID-19 includes direct viral injury due to high concentration of ACE 2 receptor on podocytes, dehydration causing pre-renal AKI, cytokine release syndrome, organ cross talk or systemic inflammation^30^,^36^. Perinephric fat stranding was the most common finding (30.6 %) that could be primarily due to systemic inflammation and increase in inflammatory markers^37^. Renal infarcts were seen in 12 % patients secondary to attenuated vessel or vascular occlusion. Some of these patients also had infarcts involving other organs. These patients required prolonged anticoagulant therapy and surgical management thus increasing morbidity in patients. In our study, renal findings especially renal infarcts were more common in patients with severe pulmonary disease (p=0.02). It was in concordance with study done by Pei G et al^38^. Urinary bladder wall thickening with or without peri vesical fat stranding suggesting cystitis was also seen. Expression of ACE2 receptors in the urinary bladder predisposes patients to cystitis.

Major vascular occlusion (SMA, SMV and their branches) was seen in 16 patients (21.3 %) leading to bowel ischemia and solid organ infarcts, thus significantly increasing morbidity and mortality in patients. Vascular occlusion was seen with a significantly increased likelihood in ICU patients (OR=4.8, p=0.04) and severe COVID-19 infection (p=0.02). Findings suggestive of SARS-CoV-2 having a direct inflammatory effect on the vascular endothelium leading to endothelitis and thrombosis have been reported^39^.

The incidence of invasive mucormycosis has drastically increased in COVID-19 patients, especially in patients with impaired glycaemic contro^19^,^10^,^40^,^41^. It is a rare opportunistic infection seen in immunocompromised people. Due to its angio-invasive nature, it is one of the most lethal fungal infections. Endothelial injury, impaired blood glucose and increased free iron in COVID-19 patients favours growth of mucormycosis^40^,^41^. Although rhino orbital involvement is most common manifestation of mucormycosis, abdominal involvement was also noted in the form of bowel wall ischemia & renal arterial thrombus mediated renal infarct. In our cohort, 6 patients with renal infarcts and 5 patients with bowel ischemia secondary to vascular thrombosis showed broad aseptate, hyaline ribbon like right angled branching fungal hyphae s/o mucormycosis on histopathological examination performed on operated specimen. The debridement surgery needs to be performed as soon as possible before infection spreads to adjoining areas. Use of combination antifungal therapy remains controversial and requires further studies for evaluation. Adjuvant therapies are also not supported strongly due to the lack of clinical trials. Prompt and aggressive treatment is required in a patient with SARSCoV-2 if coinfected with invasive mucormycosis^34^,^42^.

Except D-Dimer (p=0.01), other lab parameters i.e., Liver function test Kidney function test, IL-6 and ferritin were found to have no statistically significant association with abdominal findings in our study (Table 5 and Table 6).

**Table 5:** Summary of laboratory parameters in COVID-19

Parameter	With positive CT finding (n =65)	Without positive CT findings (n=10)	p value
Deranged LFT*	31	6	1
Deranged KFT +	28	4	0.73
D dimer#	31	6	0.01
IL-6 ^	29	6	0.17
Ferritin @	27	5	0.7

* In 64 patients with available data

+ In 66 patients with available data

# In 41 patients with available data

^ In 44 patients with available data

@ In 48 patients with available data

**Table 6:** Correlation of various systemic/organ manifestations of abdominal COVID with pulmonary severity

	Severe CTSI (>15) n = 19	Mild to moderate CTSI (</= 15) n= 56	p value
Hepatobiliary system	12	23	0.11
Pancreas	1	4	1
Gastrointestinal system	12	16	0.01
Excretory system	10	13	0.02
Vascular system	8	8	0.02
Peritoneal findings	10	16	0.09

Periodic monitoring of D-Dimers in COVID-19 patients may be used to predict the risk of abdominal complications especially ischemic complications in COVID-19 as described in past studies^26^,^27^.

The main limitation of this study was that it was a single centre study. Autopsy and pathologic correlation were not done due to government guidelines and regulations. Relationship of Mucormycosis with diabetes or overuse of steroids could not be done due to limited number of cases and non-availability of medication records.

## Conclusion

There can be a myriad of imaging manifestations in COVID-19 which can be multisystemic or predominantly affecting a single organ system. Severe abdominal features can cause significant mortality & morbidity if left undiagnosed/untreated and hence warrants early interventions. Abdominal manifestations, vascular occlusions and elevated D-dimer levels are associated with severe COVID-19 infection and ICU admission. CAM can further degrade the prognosis due to its angio-invasive nature with increased propensity to cause extensive infarcts and multi-organ damage.

## Recommendation

COVID-19 patients with abdominal symptoms should get an abdominal CT to evaluate the cause.

## References

[1] Chams N, Chams S, Badran R, Shams A, Araji A, Raad M (2020). COVID-19: A Multidisciplinary Review. Front Public Health.

[2] Kimia Vakili MF, Kimia Vakili MF (2020). Critical complications of COVID-19: A descriptive meta-analysis study. Rev Cardiovasc Med.

[3] Luo S, Zhang X, Xu H (2020). Don't Overlook Digestive Symptoms in Patients With 2019 Novel Coronavirus Disease (COVID-19). Clin Gastroenterol Hepatol.

[4] Cheung KS, Hung IFN, Chan PPY, Lung KC, Tso E, Liu R (2020). Gastrointestinal Manifestations of SARSCoV-2 Infection and Virus Load in Fecal Samples from a Hong Kong Cohort. Systematic Review and Meta-analysis. Gastroenterology.

[5] Cholankeril G, Podboy A, Aivaliotis VI, Tarlow B, Pham EA, Spencer SP (2020). High Prevalence of Concurrent Gastrointestinal Manifestations in Patients with Severe Acute Respiratory Syndrome Coronavirus 2: Early Experience from California. Gastroenterology.

[6] Goldberg-Stein S, Fink A, Paroder V, Kobi M, Yee J, Chernyak V (2020). Abdominopelvic CT findings in patients with novel coronavirus disease 2019 (COVID-19). Abdom Radiol (NY).

[7] Bhayana R, Som A, Li MD, Carey DE, Anderson MA, Blake MA (2020). Abdominal Imaging Findings in COVID-19: Preliminary Observations. Radiology.

[8] Rahman HS, Aziz MS, Hussein RH, Othman HH, Salih Omer SH, Khalid ES (2020). The transmission modes and sources of COVID-19: A systematic review. Int J Surg Open.

[9] Muthu V, Rudramurthy SM, Chakrabarti A, Agarwal R (2021). Epidemiology and Pathophysiology of COVID-19-Associated Mucormycosis: India Versus the Rest of the World. Mycopathologia.

[10] Pal R, Singh B, Bhadada SK, Banerjee M, Bhogal RS, Hage N (2021). COVID-19-associated mucormycosis: An updated systematic review of literature. Mycoses.

[11] Yuvaraj M, Mathapati PM, Seena CR, Ramaswami S (2021). Gastric mucormycosis with splenic invasion a rare abdominal complication of COVID-19 pneumonia. J Clin Imaging Sci.

[12] Horvat N, Pinto PVA, Araujo-Filho JAB, Santos JMMM, Dias AB, Miranda JA (2021). Abdominal gastrointestinal imaging findings on computed tomography in patients with COVID-19 and correlation with clinical outcomes. Eur J Radiol Open.

[13] Keshavarz P, Rafiee F, Kavandi H, Goudarzi S, Heidari F, Gholamrezanezhad A (2021). Ischemic gastrointestinal complications of COVID-19: a systematic review on imaging presentation. Clin Imaging.

[14] Barkmeier DT, Stein EB, Bojicic K, Otemuyiwa B, Vummidi D, Chughtai A (2021). Abdominal CT in COVID-19 patients: incidence, indications, and findings. Abdom Radiol (NY).

[15] Lui K, Wilson MP, Low G (2021). Abdominal imaging findings in patients with SARS-CoV-2 infection: a scoping review. Abdom Radiol (NY).

[16] Singh P, Singh SP, Verma AK, Raju SN, Parihar A (2021). A Systematic Review of Abdominal Imaging Findings in COVID-19 Patients. Visc Med.

[17] Patel RK, Chandel K, Mittal S, Tripathy T (2021). Abdominal Computed Tomography Findings among COVID-19 Patients with Index Gastrointestinal Manifestations: A Preliminary Single-center Experience. Euroasian J Hepatogastroenterol.

[18] Abdelmohsen MA, Alkandari BM, Gupta VK (2021). Gastrointestinal tract imaging findings in confirmed COVID-19 patients: a non-comparative observational study. Egypt J Radiol Nucl Med.

[19] Funt SA, Cohen SL, Wang JJ, Sanelli PC, Barish MA (2021). Abdominal pelvic CT findings compared between COVID-19 positive and COVID-19 negative patients in the emergency department setting. Abdom Radiol (NY).

[20] Tirumani SH, Rahnemai-Azar AA, Pierce JD, Parikh KD, Martin SS, Gilkeson R (2021). Are asymptomatic gastrointestinal findings on imaging more common in COVID-19 infection? Study to determine frequency of abdominal findings of COVID-19 infection in patients with and without abdominal symptoms and in patients with chest-only CT scans. Abdom Radiol (NY).

[21] Pirola L, Palermo A, Mulinacci G, Ratti L, Fichera M, Invernizzi P (2021). Acute mesenteric ischemia and small bowel imaging findings in COVID-19: A comprehensive review of the literature. World J Gastrointest Surg.

[22] Agarwal L, Agarwal A, Advani S, Katiyar V, Chaturvedi A, Madhusudhan KS (2021). The eyes see what the mind seeks: a systematic review of abdominal imaging findings in patients with COVID-19. Br J Radiol.

[23] Ojha V, Mani A, Mukherjee A, Kumar S, Jagia P (2021). Mesenteric ischemia in patients with COVID-19: an updated systematic review of abdominal CT findings in 75 patients. Abdom Radiol (NY).

[24] Taya M, Paroder V, Redelman-Sidi G, Gangai N, Golia Pernicka JS, Gollub MJ (2021). Abdominal imaging findings on computed tomography in patients acutely infected with SARS-CoV-2: what are the findings?. Emerg Radiol.

[25] Vadvala HV, Shan A, Fishman EK, Gawande RS (2021). CT angiography of abdomen and pelvis in critically ill COVID-19 patients: imaging findings and correlation with the CT chest score. Abdom Radiol (NY).

[26] Singh B, Mechineni A, Kaur P, Ajdir N, Maroules M, Shamoon F (2020). Acute Intestinal Ischemia in a Patient with COVID-19 Infection. Korean J Gastroenterol.

[27] Hosoda T, Orikasa H (2022). A fatal case of extensive gastrointestinal necrosis due to portal and mesenteric vein thrombosis in the post-acute phase of COVID-19. J Infect Chemother.

[28] Haj M, Haj M, Rockey DC (2018). Ogilvie's syndrome: management and outcomes. Medicine.

[29] Samuel SV, Viggeswarpu S, Wilson BP, Gopinath KG (2021). Acute colonic pseudo-obstruction in two patients admitted with severe acute respiratory syndrome-coronavirus-2 pneumonia. IDCases.

[30] Balaban DV, Baston OM, Jinga M (2021). Abdominal imaging in COVID-19. World J Radiol.

[31] Lei P, Zhang L, Han P, Zheng C, Tong Q, Shang H (2020). Liver injury in patients with COVID-19: clinical profiles, CT findings, the correlation of the severity with liver injury. Hepatol Int.

[32] Tafreshi S, Whiteside I, Levine I, D'Agostino C (2021). A case of secondary sclerosing cholangitis due to COVID-19. Clin Imaging.

[33] Bohn MK, Hall A, Sepiashvili L, Jung B, Steele S, Adeli K (2020). Pathophysiology of COVID-19: Mechanisms Underlying Disease Severity and Progression. Physiology.

[34] Kanmaniraja D, Kurian J, Holder J, Gunther MS, Chernyak V, Hsu K (2021). Review of COVID-19, part 1: Abdominal manifestations in adults and multisystem inflammatory syndrome in children. Clin Imaging.

[35] Liu F, Long X, Zhang B, Zhang W, Chen X, Zhang Z (2020). ACE2 Expression in Pancreas May Cause Pancreatic Damage After SARS-CoV-2 Infection. Clin Gastroenterol Hepatol.

[36] Ronco C, Reis T (2020). Kidney involvement in COVID-19 and rationale for extracorporeal therapies. Nat Rev Nephrol.

[37] Ma T, Cong L, Ma Q, Huang Z, Hua Q, Li X (2021). Study on the correlation between preoperative inflammatory indexes and adhesional perinephric fat before laparoscopic partial nephrectomy. BMC Urol.

[38] Pei G, Zhang Z, Peng J, Liu L, Zhang C, Yu C (2020). Renal Involvement and Early Prognosis in Patients with COVID-19 Pneumonia. J Am Soc Nephrol.

[39] Siddiqi HK, Libby P, Ridker PM (2021). COVID-19 – A vascular disease. Trends Cardiovasc Med.

[40] Singh AK, Singh R, Joshi SR, Misra A (2021). Mucormycosis in COVID-19: A systematic review of cases reported worldwide and in India. Diabetes Metab Syndr.

[41] Mahalaxmi I, Jayaramayya K, Venkatesan D, Subramaniam MD, Renu K, Vijayakumar P (2021). Mucormycosis: An opportunistic pathogen during COVID-19. Environ Res.

[42] Chakrabarti A, Singh S (2020). Management of Mucormycosis. Curr Fungal Infect Rep.

[43] Shiralkar K, Chinapuvvula N, Ocazionez D (2020). Cross-Sectional Abdominal Imaging Findings in Patients With COVID-19. Cureus.

